# T-cell Acute Lymphoblastic Leukemia in Crisis: Hyperleukocytosis, Tumor Lysis Syndrome, and Innovative Approaches

**DOI:** 10.7759/cureus.52017

**Published:** 2024-01-10

**Authors:** Gordon White, Mariel Duchow, Levy Amar

**Affiliations:** 1 Internal Medicine, John F. Kennedy (JFK) Medical Center/University of Miami Miller School of Medicine, Atlantis, USA

**Keywords:** leukapheresis, continuous renal replacement therapy (crrt), pulmonary critical care, hematology-oncology, t-cell leukemia, critical care nephrology

## Abstract

T-cell acute lymphoblastic leukemia (T-ALL) is a rare hematologic malignancy with a unique set of clinical challenges when it occurs in adults. This case report presents the complex management of a 32-year-old male with T-ALL who developed symptomatic hyperleukocytosis and tumor lysis syndrome. Upon presentation, the patient exhibited a constellation of critical clinical and laboratory findings, including leukocytosis, anemia, thrombocytopenia, hyperkalemia, high-anion gap metabolic acidosis, and acute kidney injury. Despite an initial diagnosis of an allergic reaction, the subsequent course of the disease necessitated rapid medical intervention and consultation with multiple specialties, including hematology-oncology and nephrology. The challenges encountered in managing this patient's condition, particularly in an intensive care unit setting, underscored the need for a tailored and multidisciplinary approach. Treatment modalities included leukapheresis, continuous renal replacement therapy, aggressive fluid resuscitation, and chemotherapy. The case highlights the intricate decision-making processes and adaptability required when addressing T-ALL with hyperleukocytosis and tumor lysis syndrome, particularly in cases where conventional chemotherapy is contraindicated. This report underscores the importance of ongoing research and the need for standardized treatment protocols for such complex clinical scenarios.

## Introduction

T-cell acute lymphoblastic leukemia (T-ALL) stands as a rare affliction within the adult demographic, presenting an incidence ranging from 10 to 17 cases per million individuals. Among the spectrum of acute lymphoblastic leukemia (ALL) subtypes in adults, T-cell-specific disease represents approximately 25% of cases [1-3]. The typical clinical presentation of ALL is marked by hematopoietic aberrations, including anemia, neutropenia, or thrombocytopenia, as the lymphocytic cells replace the normal marrow constituents, yielding symptoms encompassing fatigue, pyrexia, night sweats, weight loss, and spontaneous ecchymosis or hemorrhage [4].

A possible sequela of T-ALL is hyperleukocytosis (HL), defined by an elevated white blood cell (WBC) count exceeding 300x10^9^ cells per liter [5]. The exuberant proliferation of lymphocytes culminates in microvascular occlusion, heralding complications such as cerebrovascular accidents, pulmonary leukostasis, and acute renal failure [5,6]. The cohort of patients grappling with pulmonary leukostasis bears a mortality rate spanning 20-40% [7-9]. Furthermore, patients contending with respiratory and neurologic compromise have a staggering mortality rate of 90% within the span of one week.

The primary therapeutic modality for managing T-ALL resides in the expeditious reduction of leukocytic counts via chemotherapeutic intervention or hydroxyurea. More specifically, hydroxyurea precipitates a noteworthy reduction in WBC counts, often from 50% to 80% within a 48-hour window [10]. However, clinical scenarios may arise where the prompt initiation of chemotherapy and hydroxyurea is impeded by factors such as tumor lysis syndrome, renal insufficiency, or severe metabolic changes. Of note, tumor lysis syndrome is diagnosed when two of the following four criteria are met: potassium greater than or equal to 6 mmol/L, phosphorus greater than or equal to 4.5 mg/dL, calcium less than or equal to 7 mg/dL, and uric acid greater than or equal to 8 mg/dL [11]. In these scenarios when continuation of chemotherapy can further worsen the patient's condition, recourse to alternative strategies, specifically leukapheresis, merits consideration.

Leukapheresis has been classified by the American Society for Apheresis (ASFA) as a second-line therapeutic approach (Category II recommendation) [12]. Regrettably, a comprehensive exploration of the literature reveals a lack of evidence supporting the incorporation of leukapheresis with chemotherapy within the critical care setting. Moreover, the findings gleaned from two multicenter retrospective investigations failed to establish a statistically significant advantage regarding the combined use of leukapheresis and chemotherapy [13,14]. It is noteworthy that these investigations were confined to patients with acute myeloid leukemia (AML) and none were grounded in randomized controlled trials. Therefore, in circumstances that individuals are unable to tolerate conventional chemotherapy or hydroxyurea, the therapeutic deployment of leukapheresis stands as a notable consideration [15].

## Case presentation

We present a compelling clinical case involving a 32-year-old male patient with a pertinent medical history marked by depression and alcohol use disorder. He arrived at the emergency department via ambulance, reporting severe dyspnea. The onset of his respiratory distress was concomitant with recent antibiotic treatment prescribed for an enlarged cervical lymph node. During transit, the patient received epinephrine twice due to suspicion of an allergic reaction. On arrival, the patient displayed tachycardia with a heart rate in the range of 120 beats per minute and profuse diaphoresis. The initial laboratory assessment, as delineated in Table 1, unearthed multiple anomalies, including leukocytosis, normocytic anemia, thrombocytopenia, hyperkalemia, high-anion gap metabolic acidosis, and acute kidney injury. Arterial blood gas analysis unveiled metabolic acidosis and hypoxemia. Consequently, fibrin and D-dimer assays were conducted, raising concerns about potential coagulopathy. Radiological examinations, including chest radiography and ventilation-perfusion scanning, failed to disclose any aberrations. Despite receiving supplemental oxygen at 5 liters per minute via a nasal cannula, the patient's acidosis exacerbated, hypoxemia persisted, and clinical manifestations indicative of right heart strain emerged. Subsequently, consultations were sought from the disciplines of hematology-oncology and the intensive care unit (ICU). The patient was started on empirical antibiotic therapy and was initiated on hydroxyurea therapy before being transferred to the step-down unit. However, within a few hours, the patient clinically deteriorated, necessitating a rapid response.

**Table 1:** Laboratory findings on presentation CBC: complete blood count; WBC: white blood cells; HGB: hemoglobin; HCT: hematocrit; PLT: platelets; MCV: mean corpuscular volume; CMP: comprehensive metabolic panel; BUN: blood urea nitrogen; PT: prothrombin time; PTT: partial thromboplastin time; INR: international normalized ratio; PCO2: partial pressure of carbon dioxide; PO2: partial pressure of oxygen; O2 sat: oxygen saturation

	Study	Patient's value	Reference range	Units
CBC	WBC	>440	4-10.5	10^3^/uL
	HGB	11	13.7-17.5	g/dL
	HCT	35.6	40.1-51	%
	PLT	38	150-400	10^3^/uL
	MCV	90.4	79-92.2	FL
CMP	Sodium			
	Potassium	5.3	3.5-5.2	mmol/L
	Bicarbonate	17	19-34	mmol/L
	BUN	13	6-22	mg/dL
	Creatinine	1.69	0.43-1.13	mg/dL
	Calcium	8.1	8.4-10.2	mg/dL
	Phosphorus	4.1	2.3-5	mg/dL
	Uric acid	8.6	2.7-8.5	mg/dL
Hepatic function and coagulation markers	PT	18.8	10-12.8	Seconds
	PTT	32	25-38	Seconds
	INR	1.6	0.8-1.1	
	Fibrin	148	207-493	mg/dL
	D-dimer	12702	0-529	ng/mL
	Total bilirubin	0.9	0.1-1.2	mg/dL
Arterial blood gas	pH	7.179	7.35-7.45	
	PCO2	26.3	35-45	mmHg
	PO2	66.3	80-90	mmHg
	Base excess	-17.4	-2-2	
	O2 sat	93	85-98	%
	Comments	Room air		

During the course of the rapid response intervention, the patient's physical examination exhibited minimal alterations, albeit with pronounced somnolence. In light of this clinical deterioration, endotracheal intubation was deemed imperative for airway protection. Laboratory results now revealed a WBC count of 773x10^3^/uL, a hemoglobin level of 6.6 g/dL, a platelet count of 35x10^3^/uL, and a lactic acid level of 19.3 mmol/L. Furthermore, additional laboratory indices met the diagnostic criteria for tumor lysis syndrome as the potassium level was 6.4 mmol/L, phosphorus 6.3 mg/dL, calcium 6 mg/dL, and uric acid 11 mg/dL. Aggressive fluid resuscitation, bicarbonate infusion, rasburicase, and Solu-Medrol (Pfizer, New York, New York, United States) were expeditiously started. Hydroxyurea was promptly discontinued in light of the tumor lysis syndrome. Nephrology consultation was duly sought, and the patient was subsequently transferred to the ICU.

The patient received an array of blood products, including packed red blood cells, cryoprecipitate, and platelets. Additionally, the patient became hemodynamically unstable, necessitating three vasopressor agents, all of which were titrated to maximal effect. Flow cytometric analysis definitively confirmed a diagnosis of T-ALL. Given the patient's symptomatic HL and concomitant tumor lysis syndrome, hematology-oncology planned for the initiation of chemotherapy following a 48-hour stabilization period. In the meantime, recommendations included leukapheresis concomitant with continuous renal replacement therapy (CRRT). Within a span of 36 hours from hospital admission, the patient received eight units of packed red blood cells, three units of fresh frozen plasma, two units of cryoprecipitate, and two units of platelets. During this interval, the patient had a cumulative net positive fluid balance of 25 liters, while oliguria persisted.

Over the subsequent 48 hours, the patient oscillated between sessions of CRRT and leukapheresis. On the third day of hospitalization, the WBC count had declined to 24x10^3^/dL, precipitating the commencement of chemotherapy and the discontinuation of leukapheresis. The patient's respiratory status remained compromised due to diminished lung compliance from fluid overload, as evidenced by a plateau pressure of 35 cm H2O. Fortunately, the patient's clinical status continued to improve each day. On the eighth day of hospitalization, he was successfully extubated without incident, and the vasopressor support was weaned. Subsequently, intrathecal chemotherapy was administered on the 11th day, and the patient was transitioned to the general inpatient ward. Ultimately, the initial kidney injury and subsequent renal failure were likely multifactorial but largely as a result of acute tubular necrosis in the setting of shock. Although uric acid tubular nephropathy was not ruled out, the early use of rasburicase and resulting decrease in uric acid levels likely prevented and treated any further insult. As a result, renal function slowly recovered throughout the hospitalization, ultimately preventing the need for ongoing dialysis prior to discharge. The patient's discharge occurred on hospital day 34.

## Discussion

In this report, we described a complex clinical scenario featuring an adult patient afflicted with T-ALL, HL, and pulmonary leukostasis. Symptomatic HL in such patients portends grave illness with an unfavorable prognosis. The absence of standardized therapeutic protocols for leukapheresis can be attributed to divergent research findings, including studies that failed to definitively correlate leukapheresis with reduced mortality [13,14,16-18]. As a result, current clinical guidelines endorse primary HL management through chemotherapy [1]. Regrettably, patients grappling with tumor lysis syndrome, severe renal insufficiency, and metabolic derangements may not be suitable candidates for conventional chemotherapy or hydroxyurea, as evinced in our clinical vignette. Rios-Olias et al. retrospectively analyzed tumor lysis syndrome in ALL patients undergoing induction chemotherapy and concluded that the sole development of tumor lysis syndrome immensely increased mortality [19]. The case we presented added further complexity, considering the additional organ systems involved while also achieving the resolution of organ dysfunction and overall positive outcomes for the patient upon discharge.

Hence, the task of managing symptomatic HL, especially when concomitant multiorgan failure and tumor lysis syndrome are in play, remains a formidable challenge, compounded by the absence of a universally accepted treatment algorithm. Hölig and Moog have advanced the proposition that patients with symptomatic HL should promptly undergo leukapheresis, contingent upon the treatment facility's expertise and capacity for daily leukapheresis, until either chemotherapy commencement or a reduction in WBC count to therapeutic levels is achieved [20].

## Conclusions

We presented a rare case of T-ALL complicated by HL, pulmonary leukostasis, and tumor lysis syndrome. This case underscores the importance of clinical awareness and vigilance when managing complex hematologic malignancies. Early recognition of complications and timely interventions are critical for improving patient outcomes. T-ALL manifests infrequently in the adult population and is characterized by a clinical presentation rooted in hematologic aberrations. The hyperproliferation of lymphocytes in T-ALL predisposes patients to HL, a condition characterized by the occlusion of small-caliber blood vessels, culminating in cerebrovascular events, pulmonary leukostasis, and acute renal insufficiency. Further research endeavors are imperative to ascertain whether a specific cohort of patients burdened by symptomatic HL, who are not immediately amenable to chemotherapy or hydroxyurea, may derive therapeutic benefit from leukapheresis. Although our clinical case narrative remains anecdotal, it proffers initial insights into the potential utility of this therapeutic avenue.

## References

[1] Marks DI, Rowntree C (2017). Management of adults with T-cell lymphoblastic leukemia. Blood.

[2] Dores GM, Devesa SS, Curtis RE, Linet MS, Morton LM (2012). Acute leukemia incidence and patient survival among children and adults in the United States, 2001-2007. Blood.

[3] Sentís I, Gonzalez S, Genescà E (2020). The evolution of relapse of adult T cell acute lymphoblastic leukemia. Genome Biol.

[4] Puckett Y, Chan O (2023). Acute Lymphocytic Leukemia. StatPearls [Internet].

[5] Lee H, Park S, Yoon JH (2021). The factors influencing clinical outcomes after leukapheresis in acute leukaemia. Sci Rep.

[6] Abla O, Khanani MF, Hitzler JK (2004). Complications of hyperleukocytosis and leukapheresis in pediatric acute leukemias. Blood.

[7] van Buchem MA, te Velde J, Willemze R, Spaander PJ (1988). Leucostasis, an underestimated cause of death in leukaemia. Blut.

[8] Dutcher JP, Schiffer CA, Wiernik PH (1987). Hyperleukocytosis in adult acute nonlymphocytic leukemia: impact on remission rate and duration, and survival. J Clin Oncol.

[9] Bug G, Anargyrou K, Tonn T, Bialleck H, Seifried E, Hoelzer D, Ottmann OG (2007). Impact of leukapheresis on early death rate in adult acute myeloid leukemia presenting with hyperleukocytosis. Transfusion.

[10] Grund FM, Armitage JO, Burns P (1977). Hydroxyurea in the prevention of the effects of leukostasis in acute leukemia. Arch Intern Med.

[11] Adeyinka A, Bashir K (2001). Tumor Lysis Syndrome. StatPearls [Internet].

[12] Padmanabhan A, Connelly-Smith L, Aqui N (2019). Guidelines on the use of therapeutic apheresis in clinical practice - evidence-based approach from the Writing Committee of the American Society for Apheresis: the eighth special issue. J Clin Apher.

[13] Bewersdorf JP, Giri S, Tallman MS, Zeidan AM, Stahl M (2020). Leukapheresis for the management of hyperleukocytosis in acute myeloid leukemia-a systematic review and meta-analysis. Transfusion.

[14] Stahl M, Shallis RM, Wei W (2020). Management of hyperleukocytosis and impact of leukapheresis among patients with acute myeloid leukemia (AML) on short- and long-term clinical outcomes: a large, retrospective, multicenter, international study. Leukemia.

[15] Porcu P, Danielson CF, Orazi A, Heerema NA, Gabig TG, McCarthy LJ (1997). Therapeutic leukapheresis in hyperleucocytic leukaemias: lack of correlation between degree of cytoreduction and early mortality rate. Br J Haematol.

[16] Rinaldi I, Sari RM, Tedhy VU, Winston K (2021). Leukapheresis does not improve early survival outcome of acute myeloid leukemia with leukostasis patients - a dual-center retrospective cohort study. J Blood Med.

[17] Shallis RM, Stahl M, Bewersdorf JP, Hendrickson JE, Zeidan AM (2020). Leukocytapheresis for patients with acute myeloid leukemia presenting with hyperleukocytosis and leukostasis: a contemporary appraisal of outcomes and benefits. Expert Rev Hematol.

[18] Kuo KH, Callum JL, Panzarella T (2015). A retrospective observational study of leucoreductive strategies to manage patients with acute myeloid leukaemia presenting with hyperleucocytosis. Br J Haematol.

[19] Rios-Olais FA, Gil-Lopez F, Mora-Cañas A, Demichelis-Gómez R (2023). Tumor lysis syndrome is associated with worse outcomes in adult patients with acute lymphoblastic leukemia. Acta Haematol.

[20] Hölig K, Moog R (2012). Leukocyte depletion by therapeutic leukocytapheresis in patients with leukemia. Transfus Med Hemother.

